# State of the Art in Hygienic Quality of Food Ice Worldwide: A Ten-Year Review

**DOI:** 10.3390/microorganisms12040690

**Published:** 2024-03-29

**Authors:** Francesco Triggiano, Francesca Apollonio, Giusy Diella, Vincenzo Marcotrigiano, Giuseppina Caggiano

**Affiliations:** 1 Interdisciplinary Department of Medicine, Hygiene Section, University of Bari Aldo Moro, Piazza G. Cesare 11, 70124 Bari, Italy; francesco.triggiano@uniba.it (F.T.); francesca.apollonio@uniba.it (F.A.); giuseppina.caggiano@uniba.it (G.C.); 2 Prevention Department, Local Health Authority “ULSS 1 Dolomiti”, Viale Europa 22, 32100 Belluno, Italy; vincenzo.marcotrigiano@aulss1.veneto.it

**Keywords:** ice cube, packaged ice, food contact ice, food ice

## Abstract

Ice consumption has widely increased over the last decade. Cases of ice contamination by various microorganisms (bacteria, viruses and fungi) have been documented in the literature. In this review, we summarize the findings of selected articles on the hygienic and sanitary quality of food ice from 1 January 2013 to 31 December 2023. A total of 14 articles found via the PubMed search engine during the study period were reviewed. From the comparison between the ice produced on an industrial scale and the ice produced on a local scale in food businesses, the latter was found to be more contaminated by microorganisms. The most detected bacteria included *Escherichia coli*, coliforms, *Pseudomonas* spp., *Staphylococcus aureus*; three studies evaluated the presence of *Vibrio cholerae* and *Vibrio parahaemolyticus*; two studies highlighted the presence of viruses (Rotavirus and Norovirus). Finally, two studies detected the presence of fungi (molds and yeasts). Almost all authors of the studies argued that ice contamination also depends on the hygienic–sanitary quality of the ice-making machines. The results show that the information currently available in the literature on the hygienic–sanitary quality of ice is incomplete and that future national and international scientific studies need to be carried out.

## 1. Introduction

Ice is a food product that is playing an increasingly important role in the food industry [1]. Depending on its use, ice can be classified into edible ice and non-edible ice. Edible ice is a food matrix added to drinks and alcoholic products and is consumed directly by the consumer. Non-edible ice, on the other hand, is usually used to maintain the cold chain during transport and storage or for decorative purposes [1].

The consumption of contaminated ice can be a direct or indirect route for the transmission of pathogenic bacteria to humans. This can lead to outbreaks of gastrointestinal disease [2].

Some studies [3,4,5,6] conducted in different countries on the microbiological quality of ice used for food and drink have shown that it could cause gastroenteritis. The presence of pathogens in ice cubes may result from several factors, including contamination of the water used [4,7], poor hygiene conditions during production, mishandling [6] and/or the containers or bags used for final packaging [8].

Food ice can be contaminated by three different elements that can cause illness in the consumer in different ways: physical contaminants, represented by foreign bodies that can fall into the water during ice preparation; chemical contaminants, represented by substances that, if present in concentrations higher than those defined, can be harmful to human health; and, finally, biological contaminants, represented by living microorganisms [9].

Depending on whether ice is used as a foodstuff or only intended to come in contact with food to keep it fresh, the hygienic conditions of ice differ [10]. A study conducted in the UK by Nichols et al. [11] compared the microbial quality of edible ice used in drinks and used in contact with ready-to-eat food. The authors found that only 9% of ice used in drinks contained coliform bacteria and 1% of samples tested positive for *Escherichia coli*. In contrast, 23% of ice used for food storage contained coliform bacteria and 5% tested positive for *E. coli* contamination. This means that a potential risk to food safety and public health if the ice is in poor hygienic condition can occur, as it could contaminate the food that comes into contact with the ice, thereby transferring microorganisms to humans through the food chain.

To prevent the risk of pathogen transfer through cross-contamination during ice storage, it is useful to better understand the hygienic status of ice intended to come into contact with food.

To our knowledge, worldwide few published reviews [12] on the hygienic–sanitary quality of food ice exist; moreover, such studies are distributed unevenly throughout the world. Therefore, the aim of this study was to carry out a global review to evaluate the state of the art of the hygienic–sanitary quality of food ice in the world and to identify the gaps in knowledge on this topic. In particular, the objectives of this study were as follows: (i) to detect the microbiological contamination of ice; (ii) to evaluate the source of contamination.

## 2. Materials and Methods

A systematic review of the literature was carried out on articles published in the English language on Pubmed from all over the world in the period from 1 January 2013 to 31 December 2023 on the topic of the hygienic–sanitary quality of food ice.

The following keywords were used for the search: ice cube; packaged ice; food contact ice; food-associated ice; food ice. The exclusion criteria were as follows: reviews, minireviews, duplicates and articles not related to food ice.

## 3. Results

In total, 16 papers related to the hygienic–sanitary quality of ice were found by means of Pubmed during the period 2013–2023, but 14 were enrolled in this review. Two papers were excluded, one article because it analyzed the contamination of ready-to-use packaged food ice cubes with microplastics, while the second one evaluated the applicability of a UVC light-emitting diode (UVC-LED) to inactivate microorganisms present in ice. The selected articles are listed in chronological order in Table 1.

A total of eleven articles evaluated the presence of the main pathogenic microorganisms such as coliforms, *E. coli*, *S. aureus* and *V. cholerae*. Other studies investigated the presence of viruses (Rotavirus and Norovirus) [13] and fungi [14,15].

**Figure 1:**
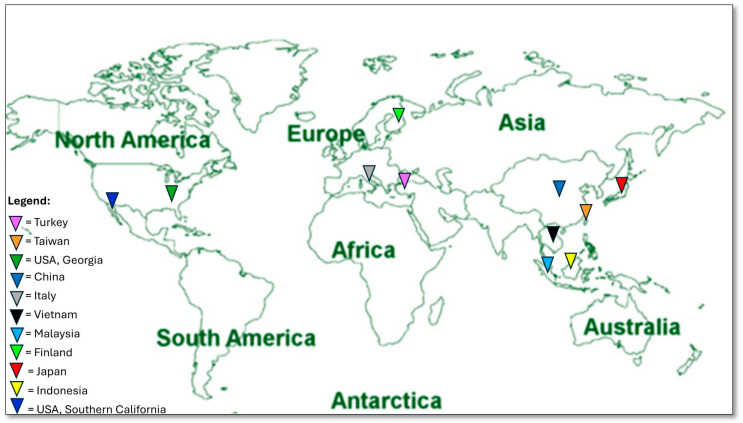
Geographic map of states that have conducted ice contamination studies.

Geographically, three studies were conducted in Italy (two in Sicily and one in the Apulian region), two in Indonesia and Vietnam and one study in each of the following countries: USA (southern California), Georgia, Turkey, Finland, Malaysia, Japan, China and Taiwan (Figure 1).

The investigation time frame shows studies carried out from 2017 onwards, with only three works carried out prior to this period.

### 3.1. Bacterial Contamination

During the period from November to December 2014, samples of water, ice cubes, frozen drinks and ice drinks were analyzed in three Indonesian cities (City A, City B and City C) to evaluate the presence of *E. coli*, *S. aureus*, *Salmonella* spp. and *Vibrio cholerae* from ice producers/manufacturers, distributors and vendors of iced beverages [16]. In particular, the presence of pathogens was assessed in three lines of drinks: with crushed ice in a plastic bag (line 1), with crushed ice in blocks (line 2) and with ice crystals (line 3). The survey was conducted through a face-to-face interview with 136 frozen drink sellers from 52 public schools, 19 ice distributors and 36 ice manufacturers in three major cities. During the sampling periods, the surfaces of ice distribution and production equipment and operator hands were also tested. The results showed the presence of *E. coli* in 6.34% of samples, of which 0.7% were confirmed as enterotoxigenic *Escherichia coli* (ETEC). In water used for the preparation of frozen drinks and ice production, *V. cholerae* was found in 0.7% of the samples, but on 2.12% of the tools used in ice distribution and production. *Staphylococcus aureus* was found on 2.02% of ice distribution and production equipment surfaces and 5.05% of production and distribution workers’ hands [16].

In particular, in City B, the ice blocks from the Line 1 dispenser were found to contain ETEC. ETECs found in ice cubes may originate from contaminated water used in ice-making. It is also possible that contaminated water has been used to wash ice from vending machines. In City C, *V. cholerae* was found in production and distribution equipment as well as in water used for ice block production. The presence of *V. cholerae* in the filtration system can contaminate the water used to make the ice blocks. *Salmonella Typhimurium* was found in one sample (1.4%), in particular, in the water used for the production of ice blocks and in ice blocks produced in City C. *Staphylococcus aureus* was found on the hands of workers producing and distributing ice in City A, as well as on ice dispensing equipment in Cities A and B [16]. The authors conclude their study by underlining that food safety practices must be implemented by manufacturers, distributors and retailers to prevent the contamination of ice [16].

Only Waturangi et al. [1] assessed the presence of *V. cholerae* in edible ice samples. Ice samples were collected from five areas of Jakarta, Indonesia. A total of 98 *V. cholerae* strains were isolated from the 40 ice samples collected from street vendors, indicating poor water quality. Serological tests showed that the majority of isolates belonged to the non-O1 serogroup (78%). The use of this contaminated water by street vendors is, therefore, a serious public health problem. Most strains were resistant to ampicillin, streptomycin, kanamycin, sulfamethoxazole–trimethoprim, erythromycin, tetracycline and ciprofloxacin. 

A study [17] conducted in the USA (southern California) compared the quality of packaged ice produced by manufacturing plants, in-store baggers (ISBs) and on-site packaged ice. Overall, 156 ice samples were analyzed for total plate count (TPC), *E. coli* and coliforms. Of the 156 samples, 120 were on-site packaged ice from convenience stores in six southern California counties: Los Angeles, Orange, San Bernardino, Riverside, San Diego and Imperial. In addition, six bags of each of two brands of International Packaged Ice Association (IPIA)-compliant manufacturer-packed ice were collected from retail outlets. Finally, a total of 24 ISB samples were collected, i.e., six ice samples from different locations for each of the four ISB brands. The results showed that 19% of the 120 on-site packaged ice samples did not meet the Packaged Ice Quality Control Standards (PIQCS) Manual [18] microbial limit of 500 most probable number (MPN)/mL and absence of coliforms and *E. coli*. Twelve of 120 (10%) samples tested positive for coliforms, and 13 of 120 (11%) samples presented high levels of TPC, even reaching values >10^4^ MPN/mL. *Staphylococcus* spp. were found in 41/120 sample (34%) of the on-site packaged ice samples, likely due to ice packing machine contamination. None of the ISB packaged and manufactured ice samples had unacceptable microbial levels. All samples investigated were free from staphylococcus spp. and *Salmonella* spp. These data suggest that there is a need for enforcement of processing regulations when ice is packaged on site [17].

Gaglio R et al., 2017 [19] evaluated the microbial quality of ice cubes from ice producers located within the Palermo province (Sicily, Italy). The study was based on the analysis of ice cubes produced at three different production stages: low volume (home production), medium volume (restaurant/bar/pub level) and high volume (industrial level). The water supply for all the ice production sites was the municipal source. This study was performed when the Legislative Decree no. 31/2001 [20], transposing Directive 98/83/EC on the quality of water intended for human consumption, went into force. The results of this study showed that all ice samples contained bacteria belonging to the Enterobacteriaceae family. Enterococci were found in two out of five ice samples from ice machines in bars and pubs, in three out of five ice samples from industrial ice machines and in none of the ice samples from domestic production. Finally, coliforms were found in all five ice samples from bar/pub ice machines, only one domestic sample and no industrial samples. These analyses were repeated two months later and showed a similar positive result, indicating a link between this problem and poor hygiene and sanitation over time. The three production systems (homemade, restaurant and industrial) showed important differences in the contamination levels of the ice samples. In particular, ice from bars and restaurants was found to have the highest levels of contaminants compared to ice from the other two groups considered to be potentially dangerous to humans.

A study [15] was carried out in the Apulia region (Italy) to evaluate the hygienic–sanitary quality of ice samples produced in public and collective catering establishments in a large area of the south of the country, analyzing the mandatory parameters (*E. coli*, coliform and Enterococci) and some optional parameters (*S. aureus*, *P. aeruginosa* and fungi) laid down in Legislative Decree no. 31/2001 [20]. Among the 99 ice samples analyzed, 54 (54.5%) were found to be in compliance with Legislative Decree no. 31/2001 [20]. The remaining 45 (45.5%) did not comply with the mandatory parameters. Specifically, 82.2% of samples contained coliforms, 40% Enterococci and 24.4% *E. coli*. Among the latter, 43 (95.5%) samples also tested positive for the additional parameters: 40% contained *P. aeruginosa* and 6.7% contained *S. aureus*. The authors conclude that the presence of these microorganisms can be attributed to poor hygienic conditions during the production and/or administration phase and to incorrect procedures for disinfection and routine maintenance [15].

A study [2] carried out in Turkey evaluated the microbiological quality of ice, the water used for ice production and the hygienic conditions of ice machines in different food establishments. A total of 105 water and ice samples were sampled from 75 restaurants/fast food establishments, 20 bars and 10 fish markets. The analyses were aimed at detecting *E. coli*, coliforms, Enterococci and psychrophilic and total aerobic mesophilic bacteria (TAMB). In addition, the microbiological contamination of the surfaces of the 105 ice-making machines was also assessed.

The results highlighted the presence of *E. coli* in 7/105 (6.7%) ice samples, while it was absent in all water samples analyzed. Instead, *E. coli* was found in 23/105 (21.9%) ice machine surface samples. Coliforms were detected in 13/105 (12.4%) water samples, 71/105 (67.6%) ice machine surfaces and 54/105 (51.4%) ice samples. Psychrophilic bacteria were detected in 83/105 (79.0%) ice machines and in 68/105 (64.7%) ice samples, but not in water samples. Total aerobic mesophilic bacteria (TAMB) were detected in 85/105 (80.9%) water samples, 98/105 (93.3%) ice samples and all ice machine surfaces (100.0%). Enterococci were found in only 13/105 (12.4%) ice samples. The presence of indicator microorganisms such as coliforms and *E. coli* in the ice samples indicated undesirable hygiene conditions.

Liao et al. [10] recently investigated in China the hygienic status of food contact ice from farmers’ markets, local supermarkets and restaurants by assessing the total bacterial count (TBC), coliform count and pathogenic bacteria contamination levels (*S. aureus*, *V. parahaemolyticus*, *Salmonella* spp., *Listeria monocytogenes*, *Shigella*). A total of 171 samples of ice used for food preservation were collected and analyzed. Overall, 128 were represented by ice samples (74.85%) used for the preservation of aquatic products; 27 (15.79%) and 16 (9.36%) were ice samples used for the cooling of poultry and livestock meat, respectively. Ice samples used for the storage of aquatic products had an average TBC of 4.88 log_10_ colony-forming units (CFU)/g and coliforms were present in 123 out of 128 (96.09%) ice samples. The TBC of 27 poultry chilling ice samples had an average level of 4.18 log_10_ CFU/g and all of these poultry chilling ice samples had coliforms. Finally, ice samples exposed to beef had a TBC of 6.11 log_10_ CFU/g and coliforms were present in 15 out of 16 ice samples. A high prevalence of *S. aureus*, *Salmonella* spp., *V. parahaemolyticus* and *L. monocytogenes* was found in ice used to preserve poultry and aquatic products. High detection rates of *S. aureus* were found in ice samples used for the preservation of aquatic products (62 samples, 48.44%). Seven samples (5.47%) were contaminated by *Salmonella* or *V. parahaemolyticus*, while two samples (1.56%) were contaminated by *L. monocytogenes.* There was no evidence of Shigella in any of the samples. With regard to ice used for the preservation of poultry meat, the prevalence of food-borne pathogens was as follows: *S. aureus* (13 samples, 48.15%), *V. parahaemolyticus* (three samples, 11.11%), *Salmonella* spp. (two samples, 7.41%). None of the samples contained *L. monocytogenes* or Shigella. In samples of ice used to preserve meat from livestock, they highlighted the presence of *S. aureus* and Salmonella in only one sample. The presence of *V. parahaemolyticus*, *L. monocytogenes* and *Shigella* was not detected in these ice samples. 

Jalava et al. [21] reported a gastrointestinal outbreak following a Christmas dinner on 9 and 10 December 2016 involving 154 people in Finland. A retrospective study was therefore carried out. In total, 24/91 people had viral gastrointestinal symptoms and the genogroup I norovirus was detected in the fecal samples of three patients. They gave each study participant a questionnaire to collect information on the type and quantity of all food and drink consumed, with particular attention to fresh produce, water and ice in drinks. The detection of norovirus genogroup I in the case patients suggests a waterborne route of transmission. This genogroup is often associated with transmission via food or water. Fecal samples from the study participants, samples of water and ice, and samples of the air ventilation system were analyzed. Water and ice samples were analyzed for *E. coli*, coliforms and total microorganisms at 22 °C. The results showed high levels of heterotrophic bacteria (estimated levels 7000 and 7500 CFU/mL, limit of detection < 3000 CFU/mL) in ice samples. No *E. coli* or coliforms were detected. No norovirus was detected in the tap water sampled in the room where the ice machines were located. The same authors hypothesized that the most likely cause of the epidemic was a faulty air valve in the ventilation system. The lid was loose, and the sealant was not properly installed. Leaky ventilation valves may be an overlooked route of transmission in gastrointestinal virus outbreaks.

From January to September 2019, Tuyet Hanh T.T. and Hanh M.H. [22] conducted a study in Vietnam assessing the food safety conditions and sanitary quality of edible ice. The food safety assessment was conducted in all 45 provincial ice production plants. Specifically, six sites only produced tube ice, five sites produced cubes (one sample taken per site) and thirty-four sites produced both tube and cube ice (one sample of each type taken per site). In total, 79 ice samples (including 39 ice cubes and 4 ice tubes) were collected. Overall, 41/79 (51.9%) ice samples were found to be polluted. In particular, 39/79 samples (49.4%) showed *E. coli* contamination and 10/79 samples (12.7%) had total coliform contamination. There was no evidence of Streptococci or *P. aeruginosa.* The study also highlights the poor hygienic quality of the ice-making machines and the premises themselves, which can lead to ice contamination.

In 2014, Mahat et al. [23] randomly collected ice samples from 30 permanent food retailers in Taman University, Johor Bahru. Tap water and bottled mineral water (as a control) were also analyzed. The samples were analyzed in order to assess the presence of fecal coliform bacteria. These microorganisms were detected in 11/30 ice cube samples (36.67%). They ranged from 1 CFU/100 mL to >50 CFU/100 mL. Ice samples from two food shops were highly contaminated with fecal coliform (>50 CFU/100 mL). Samples of the tap water and of the bottled mineral water were negative. Therefore, the same authors suggest that the ice samples were not contaminated by treated tap water or bottled mineral water, but probably by contamination during the manufacturing process. 

Nakayama et al. [24] investigated the frequency of edible ice contamination in Vietnam and Japan with extended-spectrum β-lactamase-producing *Escherichia coli* (ESBL-E) and whether contaminated ice was responsible, at least in part, for the transmission of ESBL-E among the Vietnamese population. Between March 2014 and March 2016, a total of 88 ice samples were collected and analyzed: 62 from Vietnamese restaurants and 26 from Japanese restaurants. A total of 119 bacteria capable of growing on agar containing cefotaxime (BG-CTX) were isolated from 59 (95%) restaurants in Vietnam. Six BG-CTX strains (15%) were isolated from four of the restaurants tested in Japan. ESBL production was confirmed by the disk diffusion method using CTX and CAZ, with and without clavulanic acid, as recommended by the Clinical & Laboratory Standards Institute (CLSI). Subsequently, PCR was carried out for the genotyping of the bla_CTX-M_ genes. Edible ice was significantly more likely to become contaminated in Vietnam compared to Japan. Strains isolated from ice from Vietnamese restaurants included six genera of Gram-negative bacteria: *Acinetobacter* spp., *Pseudomonas* spp., *Stenotrophomonas* spp., *Enterobacter* spp., *Aeromonas* spp. and *Klebsiella* spp. The most common were *Pseudomonas* spp. (48/119; 40%), *Acinetobacter* spp. (47/119; 39%) and *Stenotrophomonas* spp. (14/119; 12%). The genus Acinetobacter was mainly represented by *A. baumannii* (15/47, 32%) and *A. calcoaceticus* (6/47, 12.8%), whereas Pseudomonas was mainly represented by *P. putida* (9/48, 19%), *Pseudomonas* spp. (8/48, 17%) and *P. aeruginosa* (4/48, 8%). All 14 *Stenotrophomonas* strains were identified as *S. maltophilia*. Ice samples collected in Japan contained strains of the genus *Acinetobacter* (5/6) and *Pseudomonas* (1/6). Of the *Acinetobacter* strains isolated, two were identified as *A. baumannii* and one was identified as *A. junii.* Of these strains, 10% exhibited the ESBL phenotype. Surprisingly, when extended-spectrum β-lactamase—producing bacteria (ESBL-B) from contaminated ice were given to mice, ESBL-E strains emerged in their intestines. Thus, consuming contaminated edible ice may represent a significant risk factor for emerging and colonizing humans with ESBL-B.

Between mid-August and October 2012, in the state of Georgia a study was conducted [25] on the microbiological quality of ice cubes by comparing ice produced and packaged in retailers and self-service vending machines with ice produced by manufacturers monitored by the International Packaged Ice Association [26]. A total of 275 ice cube samples were analyzed, 250 from retailers and self-service machines and 25 from manufacturing companies. The 250 ice cube samples consisted of 149 retail and convenience store samples and 101 ice vending machine samples, taking a sack per site. Petrol stations, food service franchises and liquor stores represented the types of food establishments where the packaged ice was purchased. The following parameters were analyzed: heterotroph count, coliforms, non-pathogenic *E. coli*, Enterococci, *Salmonella* spp. and *L. monocytogenes*. It was found that 6% of ice samples from retailers and ice vending machines had unsatisfactory levels of heterotrophic bacteria compared to International Ice Association limits (≥500 most probable number (MPN)/100 mL). Of those, 37% contained unacceptable levels of coliform bacteria (≥1.0 MPN/100 mL), 1% contained non-pathogenic *E. coli* and 13% contained Enterococci (≥1.0 MPN/100 mL). One sample tested positive for the presence of *Salmonella* spp. Another sample tested positive for *Enterobacter agglomerans*. The microbiological quality of the ice produced in the manufacturing plants was better than that of the ice produced in the retailers and in the self-service ice machines.

### 3.2. Fungal Contamination

A study conducted in Sicily (southern Italy) in 2018 [14] investigated the presence of fungi in ice cubes produced at home, in bars and pubs and in industrial production plants and their survival in alcoholic and non-alcoholic beverages. From the 60 ice samples analyzed, nine species of yeasts and filamentous fungi were isolated. In particular, in ice from domestic production, *Cystobasidium slooffiae*, *Metschnikowia* spp. and *Meyerozyma guilliermondii* were found among the yeasts and *Hansfordia* spp. and *Penicillium glabrum* among the moulds. In ice cubes collected from bars and pubs, yeasts included *Candida intermedia*, *Cryptococcus curvatus*, *Pichia guilliermondii* and *Yarrowia lipolytica*, while filamentous fungi included *Paecilomyces lilacinus*, *Phoma leveillei*, *Purpureocillium* spp. and *Thanatephorus cucumeris*. Finally, in industrially produced ice samples, *Candida parapsilosis* and *Rhodotorula mucilaginosa* were isolated as yeast and *Fusarium* spp., *Fusarium solani* and *Paecilomyces* spp. as fungi. The survival of yeast (*Candida parapsilosis*) and filamentous fungi (*Cryptococcus curvatus*) in ice samples of alcoholic and non-alcoholic beverages was also evaluated. All strains remained viable, indicating no effects in the presence of soft and alcoholic beverages.

Caggiano G. et al. [15] also investigated the presence of fungi in ice samples collected from tourist accommodations in the Apulia region. Overall, fungi were identified in 95.8% (95/99) of the samples. In particular, filamentous fungi and yeasts were detected in 46.3% (44/95) and 20.0% (19/95), respectively. Mixed fungi were detected in 32 samples (33.6%). The main filamentous fungi identified were *Aspergillus* spp. (42.3%), *Penicillium* spp. (17.4%), *Cladosporium* spp. (16%), *Fusarium* spp. (6%), *Paecilomyces* spp. (6%) and *Alternaria* spp. (3%). Among the yeast pathogens, the following were identified: *Candida humicola* (29.4%), *Rhodotorula mucilaginosa* (20%), *Candida lipolytica* (17.9%), *Candida inconspicua* (12.6%), *Candida intermedia* (8.4%), *Saccharomyces cerevisiae* (6%) and *Candida lusitaniae* (5.2%).

### 3.3. Viral Contamination

During a school trip to Kenting, southern Taiwan, more than 200 high school students developed acute gastroenteritis after staying overnight and having breakfast at a resort on 4 and 5 March 2015 [13]. The predominance of vomiting in the clinical picture led to suspicion of norovirus gastroenteritis. As an outbreak of gastroenteritis was also reported by another group of students from a different school who stayed and ate at the same resort, an outbreak investigation was carried out to identify the source and possible carrier of the pathogen. In total, human samples were collected from ten waiters and waitresses, eight cooks and twenty-five ill students. Five samples of vomit and eleven samples of stool from the ill students tested positive for norovirus. GII.17 was the predominant genotype (13 out of 16 students, 80%). Food analyses were not carried out, as there were no leftovers from the breakfasts on the 4th and 5th of March available for testing. A total of 17 water samples were tested from the kitchen, ice machine water source and ice (source A) from other departments’ underground water system (source B). Two water samples tested positive for norovirus, one from the tap in the kitchen sink on the first floor (from source A) and the other from the outlet of the groundwater pipe (from source B). In addition, several norovirus variants, including the GI.2, GI.4 and GII.17 genotypes, were found in the ice in the ice machine and in the unboiled water before and after the ice machine filters. Phylogenetic analyses showed that noroviruses identified in ice, water and human samples clustered in the same genotypes. The authors concluded that the outbreak was caused by ice made from unboiled, inadequately filtered water contaminated with norovirus (predominant genotype GII.17).

**Table 1:** Studies on ice included in this review.

References	Country	Ice Type	Investigated Microorganisms	Main Outcomes
Waturangi et al., 2013 [1]	Jakarta, Indonesia	Edible ice	*Vibrio cholerae*	The presence of *V. cholerae* in samples of edible ice that is resistant to most antibiotics.
Mako et al., 2014 [25]	Georgia	Ice cubes from outlets, self-service machines and manufacturing companies	Heterotroph count, coliforms, non-pathogenic *Escherichia coli*, Enterococci, *Salmonella* and *Listeria monocytogenes*	Detection of unacceptable levels of heterotrophic bacteria, coliforms, non-pathogenic *E. coli* and Enterococci in ice cube samples from retail outlets and ice vending machines.
Mahat et al., 2015 [23]	Taman University, Johor Bahru, Malaysia	Ice cubes	Coliforms	Coliforms found in ice samples but not in water samples analyzed, suggesting that contamination may occur during production.
Cheng et al., 2017 [13]	Kenting, Taiwan	Ice cubes	Rotavirus and norovirus	Ice made from unboiled water contaminated with norovirus caused the outbreak and the predominant genotype was GII.17.
Gaglio et al., 2017 [19]	Sicily, Italy	Ice cubes produced in domestic freezers, bar and pub ice machines and industrial ice plants.	Enteric bacteria	Ice cubes produced at different levels are vectors of living enteric bacteria.
Hampikyan et al., 2017 [2]	Turkey	Ice cubes	*Escherichia coli*, coliforms, Enterococci, psychrophilic and total aerobic mesophilic bacteria (TAMB)	Presence of microorganisms such as coliforms, *E. coli* and Enterococci in ice samples.
Lee et al., 2017 [16]	Southern California, USA	Manufactured, in-store bagged and on-site packaged ice	Total plate count, *Escherichia coli*, coliforms	On-site packaged ice had unacceptable levels of total plate counts, coliforms and Staphylococci, while in-store bagger ice and manufactured ice were found to be free of coliforms and staphylococci and had acceptable levels of total plate counts.
Nababan et al., 2017 [17]	Indonesia	ice cubes, frozen drinks and samples of frozen ice drinks	*Escherichia coli*, *Staphylococcus aureus*, *Salmonella* spp. and *Vibrio cholerae*	Presence of enterotoxigenic ETEC, *V. cholerae* and *S. Typhimurium* in iced-drink processing stages
Nakayama et al., 2017 [24]	Vietnam and Japan	Edible ice	Extended-spectrum β-lactamase- producing bacteria (ESBL-B)	In about 95% of the Vietnamese restaurants, the edible ice was contaminated with bacteria capable of growing on agar containing cefotaxime (BG-CTX) strains, with *Acinetobacter* spp., *Pseudomonas* spp. and *S. maltophilia*.
Francesca et al., 2018 [14]	Sicily, Italy	Ice cubes produced at home, in bars and pubs, and in industrial production plants	Yeasts and filamentous fungi	In all samples, nine species of yeasts and filamentous fungi were isolated. No reduction/increase in the fungal load in the presence of non-alcoholic and alcoholic drinks.
Jalava et al., 2018 [21]	Finland	Ice cubes from public and collective catering	*Escherichia coli*, coliforms, and total bacterial count	High levels of heterotrophic bacteria in ice samples and the probable cause of the Genogroup I Norovirus gastrointestinal epidemic was a faulty ventilation valve in the ice machine room.
Caggiano et al., 2020 [15]	Apulia, Italy	Ice cubes from public and collective catering	*Escherichia coli*, coliforms, Enterococci, *Staphylococcus aureus*, *Pseudomonas aeruginosa* and fungi	*E. coli*, coliform, *P. aeruginosa*, *S. aureus*; 95% of ice samples were positive for fungi, yeasts and moulds
Tuyet Hanh T.T. and Hanh M.H., 2020 [22]	Binh Phuoc Province, Vietnam	Edible ice	Streptococci fecal, *Pseudomonas aeruginosa*, spores of sulfite-reducing anaerobes, coliforms and *Escherichia coli.*	The ice samples tested showed the presence of *E. coli* and coliforms.
Liao et al., 2023 [10]	China	Non-edible ice	Total bacterial counts, coliform counts, *Staphylococcus aureus*, *Vibrio parahaemolyticus*, *Salmonella*, *Listeria* *monocytogenes*, *Shigella*	Coliform bacteria present in over 90% of ice contact samples and high levels of *S. aureus* followed by *Salmonella*, *V. parahaemolyticus* and *L. monocytogenes*.

## 4. Discussion

This review shows the presence of few studies evaluating ice contamination worldwide to date. Despite this evidence, all the reviewed studies emphasize microbial contamination attributable to gastrointestinal pathologies. It seems, therefore, that the attention of public health aimed at investigating the quality of this food matrix can be attributed only to cases of food-borne diseases or water-borne diseases. In reality, multiple actions can be taken on a preventative level to make this food safer.

Several studies [11,27,28] have reported that microbial contamination of ice cubes is likely to be due to contaminated water supplies, inadequate manufacturing equipment and facilities and unhygienic practices.

Environmental contaminants such as ambient air and equipment (ice clips, buckets, etc.) should be considered important potential sources of ice contamination. For example, food business operators (FBOs) working in restaurants typically store ice in uncapped buckets and refrigerators, and this circumstance is conducive to environmental contamination. Furthermore, FBOs may not adequately be trained in good practices for personal hygiene, implicitly contributing to the contamination of ice with enteric bacteria [2].

To the best of our knowledge, the number of articles published on this topic worldwide is very small. For example, in Italy, a country where ice consumption is widespread in the summer season, only three studies were carried out.

Although not all articles specified the use of the ice (edible or non-edible), the majority of samples tested showed bacterial, fungal and viral contamination.

All the studies reviewed showed that ice could not be considered a safe food because it contains coliform bacteria, Enterococci, *S. aureus*, *Salmonella* spp., *Listeria* spp., fungi and viruses (Norovirus) in almost all investigations.

In addition, studies [16,17,25] comparing the microbial quality of locally and industrially packaged ice showed that the former was more contaminated. This evidence can be attributed to the difficulty of FBOs operating in small artisan businesses to implement all those preventive and management actions aimed at reducing microbial contamination, including an accurate preventive risk assessment, the choice of suitable biocides for the surfaces intended to be sanitized, procedures that include adequate cleaning frequency and targeted training.

Most articles investigating the quality of ice produced by ice machines concluded that microbiological contamination of the ice was related to poor machine hygiene. In fact, Hampikyan et al. [2] assessed the microbiological contamination of surfaces and highlighted the presence of *E. coli* and coliforms. In contrast, the water samples analyzed highlighted the presence of coliforms only. Therefore, the contamination of the ice does not appear to be related to the water, but rather to the ice-making machines in whose circuit, where correct maintenance does not take place, contamination occurs.

Another emerging aspect is that the contamination rate of fungi in edible ice can be much higher than that of bacteria. Current knowledge of fungi has shown that even the most extreme cold habitats are home to enormously diverse and metabolically active fungal communities, which make up a large part of the biodiversity at low temperatures [29]. Cold-adapted fungi are members of different phyla, and in order to survive in harsh environments, fungal strains have developed numerous strategies and functions [29].

In the future, researchers will need to better focus these studies in order to investigate the prevalence of ice contamination in different countries around the world and the main causes of this contamination. In addition, national and local health authorities need to develop specific regulations on ice contamination. All food business activities must include microbiological tests of ice and ice-making equipment. In particular, these regulations must identify and describe the methods, timing and parameters to be sought to control the hygienic–sanitary quality of ice and production machines and the methods adopted for ice machine sanitization.

Further studies are needed to evaluate the best strategies to adopt in order to reduce the microbiological contamination of ice and to ensure the maintenance of its hygienic quality. To improve the process, a HACCP (Hazard Analysis and Critical Control Points) plan for ice production could certainly be adopted, defining how to sanitize the systems, which parameters to check and how many times in a defined period. For example, the machines should be periodically disinfected with sodium hypochlorite (NaClO) in such quantities that the final concentration does not exceed the reference value set by the various countries. Furthermore, it would be advisable to use sterile containers and tools for sampling to reduce exogenous contamination. Therefore, regardless of the existence of regulations, analyses should be carried out for fecal parameters as indicators of process hygiene. It would also be advisable to evaluate the chemical composition of the ice in order to assess its influence on microbiological contamination and the possible presence of residues of disinfectants used in the sanitization of the systems.

Lastly, it is particularly important that FBOs involved in public and collective catering activities benefit from adequate training in hygiene practices, including in the food environment. There was a lack of both knowledge and good practices to prevent microbial contamination of frozen products. For example, Tuyet Hanh T.T. and Hanh M.H. [22] highlighted in their study that workers involved in ice production are mainly seasonal and most of them have an inadequate training in food safety and personal hygiene, and this may be another important source of enteric microbial contamination of ice.

## 5. Conclusions

Although ice plays an important role in the food industry in maintaining low temperatures for the preservation of food quality, the studies presented have shown that it can also be a risk factor for gastrointestinal pathologies.

In the field of public health, it is important to protect people’s health through the adoption of adequate preventive actions, to be adopted at all stages of the food chain. Preventing food-borne diseases is the task of the two main actors involved in food safety: the workers responsible for preparation and handling and the healthcare workers involved in official controls for food safety. Being aware of the microbiological hazards associated with ice as a food matrix undoubtedly contributes to minimizing the risks associated with the use and consumption of this food. The strategies to be adopted at multiple levels, national and local, must be induced by legislators and stakeholders to guarantee safer food, in light of the findings that have so far emerged, which are not without critical issues.

## Data Availability

Not applicable.
